# Home Is Not Always Safe: Pediatric Unintentional Home Injuries in a Tertiary Emergency Department Setting

**DOI:** 10.3390/jcm14207444

**Published:** 2025-10-21

**Authors:** Göksel Vatansever, Erkan Şimşekli, İdil Sivaslı, Ayşe Elçin Özge, Ahmet Hakan Aksu, Adnan Barutçu, İhsan Özdemir, Emre Yaşar Karbancıoğlu, Gülnur Göllü, Ufuk Ateş, Betül Ulukol, Tanıl Kendirli, Deniz Tekin

**Affiliations:** 1 Department of Pediatrics, Ankara University School of Medicine, 06230 Ankara, Türkiye; 2 Ankara University School of Medicine, 06230 Ankara, Türkiyesivaslidil@gmail.com (İ.S.);; 3 Division of Social Pediatrics, Department of Pediatrics, Çukurova University School of Medicine, 01330 Adana, Türkiye; adnan_barutcu@hotmail.com; 4 Division of Pediatric Emergency Medicine, Department of Pediatrics, Ankara University School of Medicine, 06230 Ankara, Türkiyeemrekarban@gmail.com (E.Y.K.);; 5 Department of Pediatric Surgery, Ankara University School of Medicine, 06230 Ankara, Türkiye; drggollu@yahoo.com (G.G.);; 6 Division of Social Pediatrics, Department of Pediatrics, Ankara University School of Medicine, 06230 Ankara, Türkiye; 7 Division of Pediatric Intensive Care, Department of Pediatrics, Ankara University School of Medicine, 06230 Ankara, Türkiye

**Keywords:** unintentional home injuries, pediatric trauma, hospitalization

## Abstract

**Background/Objectives**: Unintentional home injuries (UHIs) are a major yet preventable cause of morbidity and mortality among children. This study aimed to analyze the epidemiological characteristics, injury mechanisms, and clinical outcomes of pediatric UHIs over a nine-year period in Türkiye. **Methods**: This retrospective study included all children under 18 years who were diagnosed with UHIs in the Pediatric Emergency Department (PED) of a tertiary university hospital between January 2016 and November 2024. Demographic data, injury mechanisms, imaging findings, surgical interventions, hospitalizations, and Pediatric Intensive Care Unit (PICU) admissions were statistically analyzed. **Results**: Among 852,090 PED visits, 12,327 (1.4%) were diagnosed with UHIs. Most patients were younger than five years (72.1%) and male (56.8%). The leading causes were falls/collisions (60.6%), burns (12.1%), and foreign body aspirations (10.4%). Hospitalization and PICU admission rates were 11.7% and 1.2%, respectively, mainly involving children aged 2–4 years. Younger age and corrosive ingestion were independent predictors of hospitalization, while burns, falls/collisions, corrosive ingestion, poisoning, and foreign body aspiration significantly increased PICU admission risk. A marked rise in both hospital and PICU admissions was observed during the COVID-19 pandemic. Two fatalities occurred following falls. **Conclusions**: Falls, burns, and foreign body aspirations were the most common causes of pediatric UHIs, predominantly affecting children aged 2–4 years. Strengthening caregiver supervision, promoting safer home environments, and implementing targeted prevention programs are essential to reduce their impact on children and healthcare systems.

## 1. Introduction

Unintentional injuries are defined as physical harm resulting from the acute transfer of mechanical, thermal, electrical, chemical, or radiational energy, or from the sudden deprivation of essential elements such as heat or oxygen. Owing to their lower body mass and developmental immaturity, children are particularly vulnerable to such injuries, which represent a major source of pediatric morbidity and mortality [1,2]. Children’s distinctive anatomical and physiological characteristics—such as a larger head-to-body ratio, thinner cranial bones, weaker neck muscles, and smaller airways—further increase their susceptibility to trauma even from minor forces. Although certain compensatory mechanisms, including flexible skeletal structures, rapid tissue repair, and higher metabolic reserves, provide partial protection, these are often insufficient to counterbalance the biomechanical vulnerability inherent to early childhood [3,4]. It is estimated that globally, between 10 and 30 million children sustain injuries annually, with over 3 million resulting in death [5,6,7,8]. The majority of these injuries are attributed to preventable causes—primarily road traffic accidents, falls, burns, drownings, and poisonings—and a significant proportion occur in the home environment [5,6,7]. Approximately 90% of all childhood injuries occur in low- and middle-income countries, where overcrowded living conditions, inadequate safety infrastructure, limited supervision, and unsafe household environments—such as unsecured balconies, floor-level cooking, and improper storage of cleaning agents or medications—contribute to this disproportionate global burden [9,10].

In the United States, approximately 2.5 million children under the age of 10 present to emergency departments each year due to injuries; of these, around 96,000 require hospitalization and approximately 3100 result in death [1]. Injuries are the leading cause of mortality among children aged 1 to 18 years [11]. Similarly, national data from Türkiye indicate that approximately 13,000 children died in 2023 as a result of accidents and poisonings, making these events the most common cause of death in this age group [12]. Many pediatric trauma cases in the country are initially managed in adult emergency departments rather than pediatric units, where imaging and trauma expertise are more readily available [13,14]. This distribution may lead to underrepresentation of pediatric-specific injury data in national statistics. Unintentional home injuries (UHIs) account for an estimated 18–25% of all injury-related incidents and are the second most common reason for pediatric emergency department (PED) visits, following traffic-related accidents [14].

UHIs are most frequently observed among children aged 0–5 years. The most common causes include falls, burns, poisonings, drowning, and foreign body aspiration. In addition, emerging mechanisms such as scooter- and bicycle-related injuries have recently gained attention as increasingly frequent sources of pediatric trauma occurring in or around the home environment [15]. Among these, falls are the leading cause, accounting for 40–60% of cases, and are more frequently reported among boys [5,10,16,17]. In Türkiye, approximately 45% of these injuries occur in the 0–6 age group [14]. Several sociodemographic factors—including low socioeconomic status, lower parental education levels, multiple siblings, young parental age, or single parenthood—are identified as significant risk factors for UHIs [16]. Despite the presence of current preventive strategies, such injuries continue to constitute a substantial portion of PED visits, hospitalizations, and pediatric intensive care unit (PICU) admissions globally [1,6]. In a study by Zia et al. [18]. conducted in Pakistan, approximately 22% of children experiencing UHIs required hospitalization, whereas a multicenter study by Ye et al. [19]. in China reported that 5.5% of such cases necessitated PICU care.

The clinical spectrum of pediatric UHIs is broad, ranging from minor contusions to life-threatening head injuries or poisoning. Symptoms and findings vary according to the mechanism: falls often cause head and extremity injuries; burns lead to airway and dermal damage; and corrosive ingestion can result in severe esophageal necrosis [20,21]. Diagnostic evaluation relies on clinical assessment and imaging, particularly radiography and computed tomography, guided by the trauma mechanism and severity [22]. Treatment is multidisciplinary, including surgical repair, airway management, decontamination, and intensive care when needed. Despite major advances in emergency medicine, prevention remains the cornerstone of reducing the burden of childhood injuries [23].

Despite a growing global awareness of childhood unintentional injuries, comprehensive national-level data encompassing the full pediatric age spectrum remain scarce in many countries, including Türkiye. Most existing studies are limited by narrow age groups, single-center data, or short study durations, which restrict the generalizability of their findings. Moreover, the COVID-19 pandemic has significantly altered patterns of domestic life, potentially reshaping the epidemiology of home-related injuries in children through school closures, mobility restrictions, and increased household confinement. Therefore, this study aimed to conduct a large-scale, multi-year analysis of pediatric unintentional home injuries in Türkiye, including the COVID-19 pandemic period, to identify key demographic and clinical patterns that can inform prevention strategies and child safety policies.

## 2. Materials and Methods

### 2.1. Study Design and Setting

A retrospective chart review was conducted for all pediatric patients aged 0 to <18 years who presented to the Pediatric Emergency Department of Ankara University Faculty of Medicine, Children’s Hospital, Ankara, Türkiye, between January 2016 and November 2024 with a diagnosis of UHIs. Ethical approval for the study was obtained from the Ankara University Faculty of Medicine Ethics Committee (Approval No: İ06-538-25).

### 2.2. Inclusion and Exclusion Criteria

All pediatric patients diagnosed with UHIs in the PED during the study period were included. UHIs were defined as injuries occurring within or around the home environment without intentional cause (e.g., falls/collisions, burns, poisonings, corrosive ingestions, foreign body aspirations, electrical or penetrating injuries).

Patients were excluded if they had incomplete medical records, presented with intentional injuries (e.g., abuse, self-harm), or sustained non-domestic trauma such as road traffic or sports-related accidents. Additionally, animal bite cases were excluded due to institutional limitations in rabies vaccine administration.

### 2.3. Patient Identification and Data Collection

Data for all included patients were extracted from the hospital information management system using a standardized data collection form. Inclusion was restricted to patients for whom specialist consultations (pediatric surgery, orthopedics, neurosurgery, plastic surgery, otorhinolaryngology, or ophthalmology) were requested, as these cases are more likely to represent clinically significant injuries. Consultation records provided specialist-confirmed diagnoses and detailed documentation, thereby enhancing data reliability within the retrospective design.

Extracted data included:

Demographics: age, sex, and year of presentation.

Injury characteristics: mechanism of injury (falls/collisions, burns, poisoning, corrosive ingestion, foreign body aspiration, penetrating/electrical trauma).

Imaging and interventions: type of imaging performed [X-ray, computed tomography (CT), ultrasonography (US), or magnetic resonance imaging (MRI)], and whether surgical intervention was required. The selection of imaging modality was determined by the attending physician’s clinical judgment based on the mechanism and suspected severity of the injury.

Clinical outcomes: hospital admission, PICU admission, and length of stay in both settings.

### 2.4. Study Population and Sampling

All patients diagnosed and treated within the specified study period were included consecutively, without random sampling, to ensure comprehensive data capture and minimize selection bias.

Statistical Analysis: Statistical analyses were performed using SPSS version 30.0 (Statistical Package for the Social Sciences). Descriptive statistics were presented as mean ± standard deviation or median (minimum–maximum) for continuous variables, and as frequency and percentage for categorical variables. Associations between the type of home injury and variables such as age, sex, surgical intervention, hospital admission, and PICU requirement were analyzed using the chi-square test or Fisher’s exact test, as appropriate. For comparisons of continuous variables, Student’s *t*-test was used when parametric assumptions were met, and the Mann–Whitney U test was used otherwise. Binary logistic regression analysis was performed to identify independent predictors of hospitalization and PICU admission among children presenting with unintentional home injuries. Variables with clinical relevance or a univariate *p*-value < 0.10 were included in the multivariate model. The dependent variables were hospitalization and PICU admission status (yes/no), while independent variables included age, gender, and injury etiology (burns, falls/collisions, corrosive ingestion, poisoning, and lower airway foreign body aspiration). Model fit was evaluated using the Omnibus test and Nagelkerke R^2^ statistics, and results were presented as odds ratios (OR) with 95% confidence intervals (CI). A *p*-value of <0.05 was considered statistically significant.

## 3. Results

**Figure 1:**
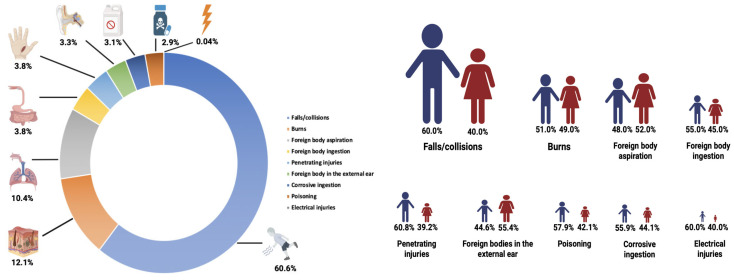
Proportional distribution of pediatric unintentional home injuries, including overall case proportions and sex-specific breakdown. The figure illustrates the relative frequency of injuries across the study population and highlights sex-related differences in injury occurrence.

During the study period, a total of 852,090 pediatric patients presented to the PED, of which 12,327 cases (1.4%) were identified as UHIs. The mean age of the patients was 4.22 years (range: 0.14–17.97), with 72.1% being under five years of age. Male children constituted 56.8% of the cohort, indicating a higher susceptibility to UHIs among boys compared to girls (*p* = 0.002) (Figure 1).

**Figure 2:**
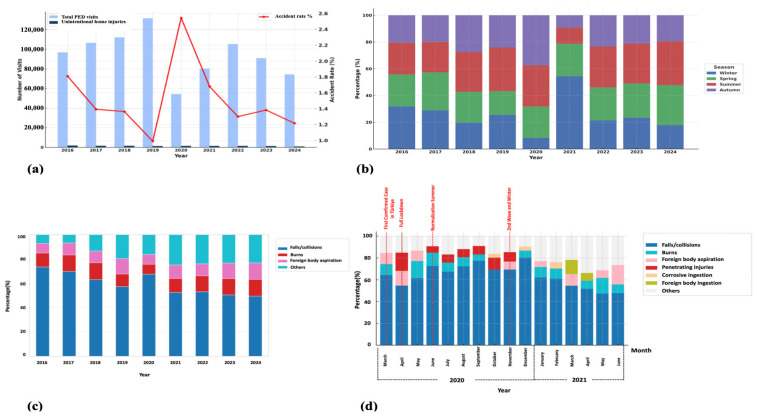
Unintentional home injuries in children: (**a**) unintentional home injuries among all pediatric emergency department visits by year; (**b**) seasonal distribution of home injuries within each calendar year; (**c**) the three most common causes of home injuries by year; (**d**) the three leading causes of home injuries during the COVID-19 pandemic period (March 2020–June 2021). PED: pediatric emergency department.

Although a declining trend in the absolute number of PED admissions due to UHIs was observed over the years, their proportional representation among all PED visits remained relatively high, particularly during the early phase of the study period. In 2020, coinciding with the onset of the COVID-19 pandemic, the total number of PED visits decreased; however, the proportion of visits related to UHIs increased, likely reflecting shifts in healthcare-seeking behavior and reduced overall PED utilization. After the relaxation of pandemic-related restrictions, the relative frequency of UHIs admissions began to fluctuate once again. A noticeable increase in the rate of UHIs was recorded during the winter months following the pandemic’s onset, compared to the same period in preceding years. Annual distribution analysis identified falls/collisions, burns, and foreign body aspiration as the most frequently reported types of home injuries. However, the frequency and ranking of these causes varied across the years. Throughout the COVID-19 pandemic, the distribution of pediatric UHIs in Türkiye evolved in line with changes in pandemic-related policies and societal dynamics. Between March 2020 and June 2021, fluctuations were particularly evident in the frequency of falls/collisions and burn cases. Starting in April 2020—marking the beginning of a nationwide lockdown—there was a noticeable increase in penetrating injuries, such as lacerations from sharp objects, which persisted through the summer normalization period and extended into the second wave of the pandemic. During the second wave, an upward trend in corrosive ingestion and foreign body ingestion was also observed. These temporal variations appear to be closely associated with factors such as mobility restrictions, school closures, lockdown measures, and seasonal transitions (Figure 2).

Falls/collisions represented the most common cause of UHIs, accounting for 60.6% of all cases. This injury mechanism was most frequently observed in children aged 2–4 years (42.6%, *n* = 3182, *p* < 0.001). Burns were the second most prevalent cause (12.1%), predominantly affecting children under two years of age (42.8%, *p* < 0.001). Foreign body aspiration was most commonly localized in the upper respiratory tract, with 68.1% of cases occurring in the 2–4-year age group (*p* < 0.001). Aspirations into the lower respiratory tract were rare and primarily observed in children under two years of age (69.4%). Foreign body ingestion was documented in 3.8% of cases, most commonly in the 2–4-year age group (48.4%, *p* = 0.006). Foreign bodies in the external auditory canal were predominantly seen in children aged 5–11 years (43.3%, *p* < 0.001). Poisonings accounted for 3.1% of cases and were most frequently observed among children aged 2–4 years (65.7%, *p* < 0.001). Ingestions of corrosive constituted 2.9% of cases, with the highest incidence in children under two years of age (46.9%, *p* < 0.001). Penetrating injuries were seen in 3.8% of patients, mostly in the 5–11-year age group (28.3%, *p* < 0.001). Electrical injuries were extremely rare (0.04%) and distributed evenly across age groups (*p* = 0.013). A total of 11.7% of patients (*n* = 1439) were hospitalized, with the majority being in the 2–4-year age group (41.1%, *p* < 0.001). Similarly, 1.2% of cases (*n* = 146) required PICU admission, with the highest proportion again observed in the 2–4-year group (45.9%, *p* < 0.001). Surgical intervention was performed in 37.9% of patients (*n* = 4666), with the highest rate observed in the 2–4-year group (49.6%, *p* < 0.001) (Table 1).

**Figure 3:**
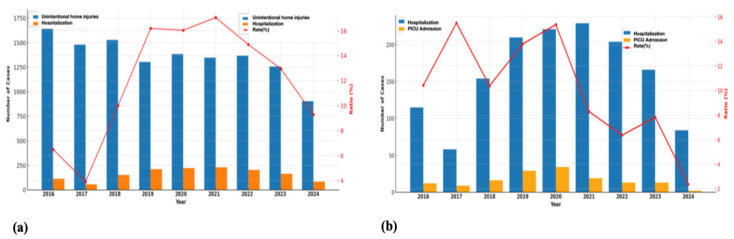
Unintentional home injuries in children: (**a**) annual distribution of hospitalizations due to unintentional home injuries; (**b**) annual distribution of pediatric intensive care unit (PICU) admissions among hospitalized cases. PICU: Pediatric intensive care unit.i.

Among the 1439 children hospitalized due to UHIs, the median age was 2.44 years (IQR: 1.46–4.33), and 55.5% were male. Throughout the study period, significant fluctuations were observed in annual hospital admission rates. Notably, during 2020—coinciding with the COVID-19 pandemic—there was a striking increase in hospitalizations due to home injuries. While admission rates remained below 10% until 2019, they rose sharply to approximately 20% in 2020 and exhibited relative variability in the following years (Figure 3). The most common cause of hospitalization was falls/collisions (34.6%), followed by poisoning (26.5%) and corrosive ingestion (17.1%). The majority of hospitalized patients were in the 2–4-year age group (41.1%). Radiological evaluations were performed in a significant proportion of cases: X-Ray in 47.7% (*n* = 687), CT in 20.7% (*n* = 298), US in 6.5% (*n* = 94), and MRI in 0.3% (*n* = 5). Surgical intervention was performed in 31.1% of hospitalized patients (*n* = 450). The median length of hospital stay was 1 day [25th–75th percentile: 1–2 days], with the maximum recorded stay being 45 days (Table 2).

A total of 146 patients were admitted to the PICU, with a median age of 2.82 years (IQR: 1.13–3.61); 58.2% were male. Notable variations were observed in the annual rates of PICU admissions. In particular, a marked increase in both the number and proportion of PICU admissions was identified in 2020, coinciding with the peak of the COVID-19 pandemic (Figure 3). The leading cause of PICU admission was poisoning (52.7%), followed by falls/collisions (34.2%) and burns (7.5%). The majority of these patients (45.9%) were in the 2–4-year age group. Among PICU patients, 11.1% underwent surgical intervention. Mechanical ventilation was required in 6.8% of cases, circulatory support in 1.4%, and intravenous lipid emulsion therapy in 1.4%. Radiologic evaluations included X-Ray in 30.1% (*n* = 44), CT in 34.1% (*n* = 50), US in 9.6% (*n* = 14), and MRI in 0.7% (*n* = 1). The median length of stay in the PICU was 1 day (IQR: 1–3) (Table 3).

Logistic regression analyses were performed to predict both hospitalization and PICU admission risks among patients presenting with UHIs. The models were statistically significant (Omnibus test, *p* < 0.001) and demonstrated acceptable goodness-of-fit (Nagelkerke R^2^ = 0.342 for hospitalization; R^2^ = 0.261 for PICU admission), with overall accuracies of 91.4% and 98.8%, respectively. Independent variables included gender, age, and causes of domestic accidents, while the dependent variables were hospitalization and PICU admission status. Each one-year increase in age was associated with approximately a 1.12-fold and 1.13-fold decrease in the likelihood of hospitalization and PICU admission, respectively. Among the causes of hospitalization, only corrosive ingestion increased the risk of hospitalization by 1.51-fold, whereas other causes were more frequently associated with discharge without hospitalization. Conversely, the risk of PICU admission increased by 16.97-fold for burns, 15.75-fold for falls/collisions, 35.46-fold for corrosive ingestion, 538-fold for poisoning, and 21.73-fold for foreign body aspiration, LRT (Table 4).

During the study period, two patients died as a result of UHIs. Both patients were male. The 16-year-old patient died from multiple organ trauma following a fall from a height, and the 9-month-old patient died from intracranial hemorrhage following a fall from the same level.

## 4. Discussion

This study adds new evidence to the existing literature by providing one of the most comprehensive, long-term analyses of pediatric unintentional home injuries in Türkiye, spanning nine years and incorporating the COVID-19 pandemic era. Unintentional home injuries remain one of the most significant yet preventable causes of trauma in children, particularly in low- and middle-income countries. In countries like Türkiye—characterized by a young population, rapid urbanization, and extended periods of indoor confinement for children—comprehensive analysis of UHIs is a public health priority. The present study, based on a large pediatric cohort over a nine-year period, offers valuable insights into the epidemiological dynamics of UHIs at the national level and contributes to the broader understanding of injury patterns in similar sociodemographic settings. Notably, the temporal variations observed during the COVID-19 pandemic underscore the influence of macro-level factors—such as mobility restrictions, school closures, and societal lockdowns—on the frequency and nature of pediatric injuries. Shifts in the prevalence of traditional injury mechanisms (e.g., falls, burns, foreign body aspiration) and the emergence of new patterns (e.g., increased penetrating trauma and poisoning) illustrate how structural changes in society can indirectly impact pediatric health outcomes. These findings directly address the study’s objectives by demonstrating how societal changes, such as pandemic-related restrictions, influenced home injury patterns and by identifying key determinants that can inform child safety and prevention policies.

Unintentional home injuries remain one of the most prevalent causes of morbidity in early childhood, particularly among children under the age of five [24]. Notably, children between the ages of two and three are at significantly higher risk due to their developmental stage and emerging independence [3]. This heightened vulnerability underscores the critical role of developmental and cognitive maturity in injury risk. Younger children are especially at risk due to heightened curiosity, increased exploratory behavior, and immature motor and cognitive abilities [25]. Previous literature consistently demonstrates that male children are disproportionately affected by UHIs [3,10,17]. This gender disparity is often attributed to a complex interplay of biological, behavioral, and psychosocial factors. Boys tend to exhibit higher levels of impulsivity, greater risk-taking behavior, and lower levels of hazard perception, all of which may predispose them to injury [25]. Our findings corroborate these observations, showing that home injuries predominantly occurred in children under five years of age—especially in the 2–4-year age group—and were more common among boys. The consistency of these trends across both high-income and low- to middle-income countries further supports the universality of age- and gender-related risk factors in UHIs. These findings highlight the need for injury prevention strategies that are both developmentally appropriate and gender-sensitive. Interventions should focus on early childhood as the most critical window for prevention and consider culturally informed approaches that address differential patterns of parental supervision and gender norms contributing to injury risk.

Although our study did not reveal an increase in the absolute number of patients diagnosed with UHI, there was a marked rise in the proportion of hospital admissions due to UHIs during the pandemic years compared to pre- and post-pandemic periods. This finding is consistent with other studies indicating that the increased time spent at home due to lockdowns led to a rise in the incidence of home injuries during the COVID-19 pandemic [13,26]. In our cohort, the most common mechanisms of injury were falls/collisions, burns, and foreign body aspiration, aligning with findings reported in both high- and middle-income countries [8,16,19,23,27,28,29]. One of the key strengths of our study lies in its nine-year coverage, encompassing the COVID-19 pandemic period, which enabled the analysis of temporal variations in injury patterns. While the distribution of UHIs remained relatively stable between 2016 and 2019, significant shifts were observed in 2020. There was a slight increase in falls/collisions compared to pre-pandemic years; however, during periods of strict lockdown and school closures—when children spent more time indoors—there was a notable rise in sharp object-related penetrating injuries and corrosive substance ingestions. These findings are consistent with previous reports by Sanford et al. [30]. in the United States and Güleryüz et al. [29] in Türkiye, both of which documented increased sharp-object injuries during lockdown periods, as well as with the study by Salman et al. [31], which highlighted a rise in corrosive substance ingestions during the pandemic. These pandemic-associated shifts may be attributed to increased time spent at home, altered parental supervision, divided attention due to remote working, and limited access to structured environments such as daycare or schools. The rise in sharp-object injuries may reflect increased unsupervised access to hazardous household items, while the increase in ingestion and aspiration incidents may be linked to the oral exploratory behavior typical of younger children being amplified by prolonged indoor confinement. These findings highlight how societal crises such as pandemics can temporarily alter the epidemiology of childhood injuries and underscore the need for adaptive prevention strategies grounded in real-time surveillance. Importantly, although total emergency visits declined during the pandemic, the proportional burden of home injuries remained elevated, emphasizing that home safety must remain a public health priority even under crisis conditions. This temporal analysis provides novel insights into how external societal factors can reshape the epidemiology of home injuries, emphasizing the need for adaptable, data-driven prevention frameworks.

Our study revealed that the mechanisms of childhood UHIs significantly differed by age group, closely aligning with developmental characteristics. Children aged 0–4 years constituted the highest-risk group. Early childhood is marked by rapid physical and cognitive development, during which children become increasingly mobile, independent, and eager to explore their environment. Since they spend most of their time at home during this period, injuries such as burns, scalds, and poisonings are more frequently observed. As children grow older and gain independence, they tend to explore areas further from home, increasing the risk of outdoor injuries [32,33]. This likely explains the relatively lower rates of UHIs in children over five years old. In our study, burns were the most common injury type among children under two years of age, emphasizing that contact with hot liquids and objects poses a serious risk during this stage. This finding is consistent with international literature, which identifies infants and toddlers as being at high risk due to immature motor control and oral exploratory behavior [5]. The 2–4-year age group was the most frequently affected cohort in our study, with falls/collisions being the predominant injury mechanisms. This is consistent with previous findings suggesting that, despite increased mobility, children in this age group lack sufficient awareness of environmental hazards [25,28,32]. These findings also suggest that parental awareness of in-home safety measures plays a critical role in injury prevention. Moreover, architectural features commonly found in Turkish homes—such as staircases, balcony openings, and the use of rugs on slippery floors—may further contribute to fall-related risks. Foreign body aspiration and ingestion incidents were also most commonly observed in the 2–4-year group, which aligns with the literature [20], likely due to the predominance of oral exploratory behaviors at this age. Poisonings were also most frequently observed in this group, whereas corrosive substance ingestions were more common among children aged 0–2 years. These patterns are consistent with findings from other studies [34,35], and underscore the ongoing public health concern posed by the accessibility of hazardous substances within home environments. Among children aged 5–11 years, foreign bodies in the external auditory canal and injuries caused by sharp or penetrating objects were observed more frequently. This may be attributed to their increased cognitive abilities coupled with reduced supervision during play and increased access to potentially dangerous items. These behaviors are likely influenced by curiosity, peer dynamics, and still-developing executive functioning skills.

In our study, 11.7% of children with UHIs were hospitalized. The most common causes requiring inpatient care were falls/collisions, poisonings, and corrosive substance ingestions. The largest proportion of hospitalized children belonged to the 2–4-year age group, indicating that this developmental period presents a particularly high risk for injury and constitutes a crucial window for targeted prevention efforts. This finding is consistent with prior literature: Gaspar et al. [36] reported a hospitalization rate of 9.9% among children and adolescents following accidents, and Wang et al. [20] identified the 1–4 age group as having the highest risk for admission due to injury. Throughout the study period, we observed substantial year-to-year variability in hospital admission rates. In 2020, coinciding with the height of the COVID-19 pandemic, there was a marked increase—doubling from under 10% in previous years to approximately 20%. This dramatic rise is consistent with studies that reported increased UHIs incidence and hospital admissions during lockdowns, likely due to more time spent at home, lapses in supervision, and delayed medical attention [29,37]. Clinically, the high proportion of imaging utilization (X-ray: 47.7%, CT: 20.7%) and surgical procedures (31.1%) reflects the significant burden of UHIs on healthcare resources. These findings further underscore the need to develop standardized pediatric imaging and triage protocols to optimize diagnostic accuracy, ensure consistency in clinical decision-making, and minimize unnecessary radiation exposure. While the median hospital stay was relatively short (1 day), some cases required extended hospitalization of up to 45 days, suggesting the potential severity and complexity of such injuries. These results emphasize the importance of early risk identification, caregiver education, and enhanced home safety practices, particularly in households with young children. Furthermore, younger age and corrosive ingestion were identified as the principal predictors of hospitalization among children presenting with UHIs. Each one-year increase in age was associated with approximately a 1.12-fold reduction in the odds of hospitalization, suggesting that developmental vulnerability in early childhood plays a critical role in determining the severity of injury outcomes. In contrast, corrosive ingestion independently increased the likelihood of hospitalization by 1.51-fold, reflecting the high tissue-destructive potential and clinical complexity of caustic exposures. These findings are consistent with previous reports that emphasize the substantial morbidity and prolonged management often required in corrosive injuries [38,39]. Taken together, the results of our analysis underscore that both age and injury mechanism are strong determinants of hospitalization, reinforcing the importance of targeted prevention strategies focused on safe chemical storage, vigilant supervision, and hazard reduction within the domestic environment.

In our study, 1.2% of children with UHIs required admission to the PICU, with poisonings being the most frequent cause, followed by falls/collisions and burns. Most of these admissions occurred in children aged 2–4 years, reflecting the developmental vulnerability and limited hazard awareness characteristic of this age group. Reported PICU admission rates in previous studies range from 0.3% to 5.5%, with falls/collisions and burns commonly identified as the leading causes, particularly among children under five [19,40]. The sharp rise in PICU admissions observed in 2020, coinciding with the peak of the COVID-19 pandemic, is consistent with earlier findings associating delayed care-seeking, reduced outpatient access, and heightened caregiver anxiety with greater injury severity [29]. Although the median PICU stay was short (1 day), the need for advanced interventions—such as mechanical ventilation (6.8%), surgery (11.1%), and intensive supportive therapies—underscores the substantial clinical burden of UHIs on pediatric critical care resources. Younger age and specific injury mechanisms emerged as significant predictors of PICU admission in our analysis. The association between younger age and higher critical injury risk aligns with previous evidence linking developmental immaturity, limited risk perception, and insufficient motor coordination to increased trauma severity in early childhood [20,41]. Etiological factors such as burns, falls, corrosive ingestion, poisonings, and foreign body aspiration markedly increased the likelihood of PICU admission, consistent with previous studies showing that traumatic, caustic, and toxic injuries frequently lead to severe clinical manifestations and a greater need for intensive monitoring and intervention [21,22,42,43]. These findings underscore the critical influence of both age and injury mechanism on the severity of UHIs and highlight the importance of proactive prevention strategies—particularly enhanced caregiver supervision, safe chemical storage, and home environment modifications—to reduce the risk of life-threatening outcomes. These results emphasize the importance of providing home safety education and developing community-based interventions targeting high-risk families.

Although mortality due to UHIs was rare in our study, the occurrence of two fatal cases highlights the potentially severe consequences of even seemingly minor mechanisms, such as same-level falls. These deaths emphasize the vulnerability of both infants and adolescents to fatal outcomes, reinforcing the importance of early prevention strategies. Prior studies have similarly reported low but non-negligible mortality rates from home injuries, particularly associated with falls from height and intracranial trauma in young children [5,44]. Given the developmental limitations in risk perception among younger age groups, targeted education and home safety modifications remain essential components of injury prevention frameworks.

Overall, this study advances current understanding by quantifying key predictors of hospitalization and critical care needs in pediatric UHIs and by providing actionable evidence to support both preventive strategies and clinical preparedness.

This study has several limitations. First, its retrospective design limited the ability to capture detailed contextual factors such as injury mechanisms, environmental circumstances, and parental supervision. Moreover, the retrospective nature of the study and the constraints of the hospital’s electronic medical records prevented consistent retrieval of information regarding the mode of transport to the hospital, the specific mechanism of collision, and the pain assessment performed in the emergency department. Second, the data were derived from a single tertiary care center, which may not reflect less severe injuries managed elsewhere or unreported cases, potentially underestimating the true community incidence. Third, the study lacked follow-up data, precluding assessment of long-term outcomes. Lastly, animal bite cases were excluded due to institutional limitations in rabies vaccine administration, which may have led to underrepresentation of certain home injuries. Despite these limitations, the study provides valuable insights into the epidemiological patterns and predictors of hospitalization and PICU admission. Future prospective, multicenter studies incorporating additional clinical variables are warranted to achieve a more comprehensive understanding of pediatric home injury dynamics and outcomes.

## 5. Conclusions

Falls/collisions, burns, and foreign body aspirations were identified as the most common mechanisms of UHIs among children, particularly in those aged 2–4 years. Corrosive ingestions and poisonings, although less frequent, were associated with significantly higher risks of hospitalization and PICU admission. These findings highlight the urgent need for preventive interventions targeting early childhood, with an emphasis on maintaining safe home environments, ensuring proper chemical storage, and promoting enhanced parental supervision. Educational programs should be directed toward parents and caregivers of preschool-aged children to improve hazard awareness and basic first-aid knowledge. Furthermore, the strong association between specific injury mechanisms and clinical severity underscores the importance of strengthening pediatric emergency preparedness and developing standardized triage and imaging protocols to optimize patient management. Although this study was conducted in Türkiye, its findings are likely generalizable to other low- and middle-income countries with similar household structures, caregiving practices, and sociodemographic characteristics. Therefore, the results can inform global child safety strategies and assist policymakers in designing context-specific injury prevention initiatives.

## Figures and Tables

**Table 1:** Characteristics of patients with unintentional home injuries (*n* = 12,327).

Characteristic	0–<2 Years		2–4 Years		5–11 Years		>12 Years		Total		*p*
	*n*	%	*n*	%	*n*	%	*n*	%	*n*	%	
Gender, male	2029	56.5	3076	58.0	1486	56.8	414	50.9	7005	56.8	0.002 *
Falls/collisions	2188	29.3	3182	42.6	1684	22.5	418	5.6	7472	60.6	<0.001 *
Burns	638	42.8	418	28.0	287	19.2	148	9.9	1491	12.1	<0.001 *
Foreign body aspiration											
URT	213	17.9	812	68.1	146	12.2	21	1.8	1192	9.7	<0.001 *
LRT	59	69.4	22	25.9	3	3.5	1	1.2	85	0.7	<0.001 *
Foreign body ingestion	113	24.1	227	48.4	109	23.2	20	4.3	469	3.8	0.006 *
Penetrating injuries	97	20.8	124	26.6	132	28.3	114	24.4	467	3.8	<0.001 *
Foreign body in the external ear	15	3.7	135	33.3	176	43.3	80	19.7	406	3.3	<0.001 *
Poisoning	100	26.2	251	65.7	31	8.1	0	0.0	382	3.1	<0.001 *
Corrosive ingestion	167	46.9	134	37.6	47	13.2	8	2.2	356	2.9	<0.001 *
Electrical injuries	2	40.0	0	0.0	1	20.0	2	40.0	5	0.0	NA
Hospitalization	568	39.5	591	41.1	221	15.4	59	4.1	1439	11.7	<0.001 *
PICU admission	62	42.5	67	45.9	14	9.6	3	2.1	146	1.2	<0.001 *
Surgical intervention	893	19.1	2316	49.6	1145	24.5	312	6.7	4666	37.9	<0.001 *

LRT; lower respiratory tract, NA; not applicable, PICU; pediatric intensive care unit, URT; upper respiratory tract. * Pearson Chi-square Test. All values are presented as *n* (%).

**Table 2:** Clinical characteristics of hospitalized patients (*n* = 1439).

Characteristic	*n*	%
Age (years) [25th–75th percentile]	2.44 [1.46–4.33] Min–Max: 0.01–17.63	
Gender, male	768	55.5
Unintentional home injuries		
Falls/collisions	498	34.6
Poisoning	382	26.5
Corrosive ingestion	246	17.1
Foreign body ingestion	96	6.7
Foreign body aspiration	95	6.6
Penetrating injuries	73	5.1
Burns	38	2.6
Others	9	0.6
Length of stay, median [25th–75th percentile]	1 [1,2] Min–Max: 1–45	

**Table 3:** Clinical characteristics of patients admitted to the pediatric intensive care unit (*n* = 146).

Characteristic	*n*	%
Age (years) [25th–75th percentile]	2.82 [1.13–3.61] Min–Max: 0.01–16.93	
Gender, male	85	58.2
Unintentional home injuries		
Poisoning	77	52.7
Falls/collisions	50	34.2
Burns	11	7.5
Corrosive ingestion	6	4.1
Others	2	1.4
Ventilation support	10	6.8
Circulatory support	2	1.4
IV lipid infusion	2	1.4
Length of stay, median [25th–75th percentile]	1 [1–3] Min–Max: 1–45	

**Table 4:** Logistic regression analysis of clinical features predicting hospitalization and PICU admission among children with UHIs.

Characteristic	Hospitalization				PICU Admission			
	OR (95% Cl Lower–Upper)		B	*p*	OR (95% Cl Lower–Upper)		B	*p*
Gender, female	0.948	(0.832–1.080)	−0.054	0.419	1.022	(0.720–1.450)	0.022	0.903
Age	0.876	(0.857–0.896)	−0.132	<0.001	0.867	(0.793–0.948)	−0.143	0.002
Falls/collisions	0.051	(0.043–0.061)	−2.972	<0.001	15.756	(2.174–114.208)	2.757	0.006
Burns	0.018	(0.013–0.026)	−4.006	<0.001	16.977	(2.186–131.830)	2.832	0.007
Foreign body ingestion	0.187	(0.144–0.244)	−1.674	<0.001				
Foreign body aspiration, URT	0.006	(0.003–0.011)	−5.159	<0.001				
Penetrating injuries	0.179	(0.134–0.239)	−1.722	<0.001				
Corrosive ingestion	1.519	(1.161–1.987)	0.418	0.002	35.465	(4.245–296.329)	3.569	0.001
Poisoning					538.654	(74.552–3891.906)	6.289	<0.001
Foreign body aspiration, LRT					21.731	(1.340–352.283)	3.079	0.030

LRT; lower respiratory tract, PICU; pediatric intensive care unit, UHIs; unintentional home injuries, URT; upper respiratory tract.

## Data Availability

Dataset available on request from the authors.

## Abbreviations

The following abbreviations are used in this manuscript:

CT	Computed tomography
MRI	Magnetic resonance imaging
PED	Pediatric emergency department
PICU	Pediatric intensive care unit
UHIs	Unintentional home injuries
US	Ultrasonography
